# Differences in joint line level and posterior condylar offset during total knee replacement with use of gap-balancing and measured resection techniques—matched cohort study

**DOI:** 10.1186/s12891-023-06722-1

**Published:** 2023-07-25

**Authors:** Bartosz M. Maciąg, Tomasz Kordyaczny, Dawid Jegierski, Marcin Łapiński, Monika Dorocińska, Krystian Żarnovsky, Grzegorz J. Maciąg, Olga Adamska, Artur Stolarczyk

**Affiliations:** Department of Orthopedics and Rehabilitation, Medical University of Warsaw, Międzyleski Specialist Hospital, Str. 2 Bursztynowa, 04-749 Warsaw, Poland

**Keywords:** Total knee arthroplasty, Gap balancing technique, Measured resection technique, Joint line level

## Abstract

**Background:**

Total knee replacement (TKR) is considered one of the most common elective orthopaedic procedures. The main focus of TKR is to offer patient's symptomatic relief from persistent knee pain. To achieve this it is crucial to restore joint biomechanics by performing proper bone cuts. Some surgeons favor the measured resection technique, others prefer gap balancing technique. The researchers of the presented study performed TKR using these two techniques. The aim of this study was to compare the postoperative change in joint line and posterior condylar offset after TKR with use of anatomic knee design implants between gap balancing and measured resection techniques.

**Methods:**

Two hundred twenty-five X-rays of patients who underwent TKR performed by a single surgeon between 2020 and 2021 were analyzed. The first group of patients (101) was operated with the use of gap balancing technique and the second group (124) was operated with the use of measured resection technique. Patients included in the study were > 50 years of age, had confirmed primary knee osteoarthritis, underwent primary TKR with a PS (posterior stabilized) knee implants without patella resurfacing and had at least 15 degree flexion contracture. T-student test and U Mann–Whitney test were used in statistical analysis of results, according to the normality of distribution examined with the Shapiro–Wilk test. Post-hoc analysis was performed using the Dwass-Steel-Crichtlow-Fligner test (DSCF).

**Results:**

The postoperative analysis showed a significantly elevated joint line level in the gap balancing group (-2.6 ± 4.1 vs -0.7 ± 4.8, *p* < 0.0005). In the gap balancing group significantly more patients had joint lines elevated > 2 mm comparing to measured resection technique. The difference between pre- and postoperative PCO (posterior condylar offset) and PCOR (posterior condylar offset ratio) results had no significant differences (100.8 ± 11.8 vs 101 ± 12.5, *p* > 0.05) between the groups.

**Conclusions:**

The results of the study suggest that when it comes to restoring joint line level measured resection technique seems to be superior in comparison to the gap balancing technique. What is more, results indicate measured resection is equal in terms of restoring posterior condylar offset to the gap balancing technique.

**Trial registration:**

NCT04164147, date of registration: November 14, 2019.

## Background

Total knee arthroplasty (TKA) is considered one of the most successful orthopaedic procedures ever. Patient satisfaction varies from 75–90% and depends on both non-surgical and surgical factors. Among non-surgical factors are socio-economic status, mental health, general physical condition, and patient’s expectations [1, 2]. One of the main goals of TKA is to offer patient's symptomatic relief from persistent knee pain. One of the factors deciding a patient's satisfaction is restoring joint biomechanics by obtaining symmetrical extension and flexion gaps. Sometimes it comes for a price of altering joint line level. On the other hand, joint line level after performing the bone cuts plays an important role in achieving stability throughout the range of motion of the knee. The surgeon has to take both aspects into account and find a golden mean between balancing the flexion/extension gaps and knee stability [3, 4].

Measured resection and gap balancing techniques are both considered good techniques for obtaining proper cuts and optimal position of the joint line. The first method relies on bony landmarks e.g. TEA (Trans-epicondylar axis), surgical TEA and Whiteside line in femoral cut while the tibial cut is performed parallel to the femoral cut so as to achieve rectangular flexion and extension gaps [5]. The gap balancing technique is based on soft tissue tension balancing being made prior to bone cuts. Measured resection technique is considered a well-known and safe method utilized for many years in TKA but there is growing evidence that gap balancing might be better in restoring native knee alignment. On the other hand, in recent systematic reviews and meta-analysis it was proved that the patient reported outcome does not differ between these techniques [6–9].

Engineers designing prostheses, wanting to meet the demanding biomechanical conditions of the knee joint, constantly introduce new models of implants. Anatomical, asymmetrically-shaped implants are innovative designs aiming to reproduce the natural shape of knee elements and override the symmetrical components in recreating joint biomechanics [10, 11]. To our best knowledge, there is a lack of papers analyzing joint line position in TKR with use of asymmetrical components in TKA. Therefore anatomical components were chosen for the procedure to further investigate this topic.

The aim of this study was to compare the postoperative change in joint line and posterior condylar offset in TKA with use of anatomic knee designs between measured resection and gap balancing techniques. The research tried to find if one of aforementioned techniques is superior in restoring preoperative level of joint line.

## Methods

This study was conducted according to The Strengthening the Reporting of Observational Studies in Epidemiology (STROBE) Statement.

Patients included in the study were > 50 years of age, had clinically and radiologically confirmed primary osteoarthritis, were undergoing primary TKR with a PS implant without patellar resurfacing and had at least 15 degree flexion contracture. Exclusion criteria included 1. Patients with prior HTO (High tibial osteotomy) or other lower limb bone surgery on the affected side in their past medical history 2. Patients with rheumatoid arthritis 3. Patients without complete radiologic examination available for review 4. Patients qualified for cruciate-retaining implants (PCL intact at time of surgery or absent flexion contracture higher than 15 degrees preoperatively) 5. Deformation equal or higher than 15 degrees of varus 6. valgus knees (hip-knee-ankle angle > 180 degrees).

A consecutive series of patients were qualified and operated by two fellowship-trained surgeons in the level III academic hospital between November 2020 and May 2021. They were operated with on-label use of PERSONA PS (Zimmer-Biomet, Warsaw, IN) total knee implants without patella resurfacing as a treatment for end-stage knee osteoarthritis. Both cohorts were operated with either gap balancing technique or measured resection, as detailed below. For both cohorts demographic patients’ data (age at surgery, sex and BMI) was generated. Demographic patients’ data are depicted in Table 1. Gap balancing patients were matched to measured resection patients.

**Table 1:** Baseline characteristics of patients included in the analysis

	Gap balancing	Measured resection	**p**
**Number of patients**	**101**	**124**	
**Mean BMI [kg/m2]**	30.27 ± 3.75	30.60 ± 3.79	0.7869
**Mean age [years]**	69.10 ± 7.91	70.72 ± 6.66	0.2709
**Side [L:P]**	55:46	63:61	0.1558
**Mean Hip-Knee Angle [degrees]**	172.09 ± 3.22	172.98 ± 2.82	0.0792

### Gap balancing technique

After knee exposure and all redundant soft tissues and osteophytes removal distal femoral and proximal tibial cuts are performed parallel to the ground line and to itself. Peripheral osteophytes are removed. Next, a dynamic tensioner—Fuzion (Zimmer-Biomet, Warsaw, IN) is placed into the knee, as in the technique described by Benazzo et al. [12]. Femoral rotation is assessed by tensioning both compartments of the knee. After a 4-in-1 cutting block placed in drilled holes, femoral rotation is once again controlled with the use of a tensioner. After confirming proper rotation all remaining femoral cuts are performed.

### Measured resection technique

After knee exposure, distal femoral cut is performed with preset value of valgus correction angle. Then in 90 degrees of flexion the 4-in-1 cutting block is placed with the surgical transepicondylar and antero-posterior (AP) axis used as reference. After femoral cuts, proximal tibia is cut parallel to the ground line, with tibial axis as reference. In case of inappropriate balance, soft tissue releases are performed.

All surgeries were performed with use of a tourniquet (average time of 60.5 min) without postoperative closed suction drainage. All surgeries were performed using a standard midline incision and medial parapatellar arthrotomy. No patella resurfacing was performed. In case of patellar arthrosis, denervation was performed. All components were implanted with the use of cement. The post-operative protocol included chemical and mechanical thromboprophylaxis unless specifically contraindicated. All patients received one dose of parenteral antibiotics at the induction of anaesthesia and two further doses post-operatively.

**Fig. 1:**
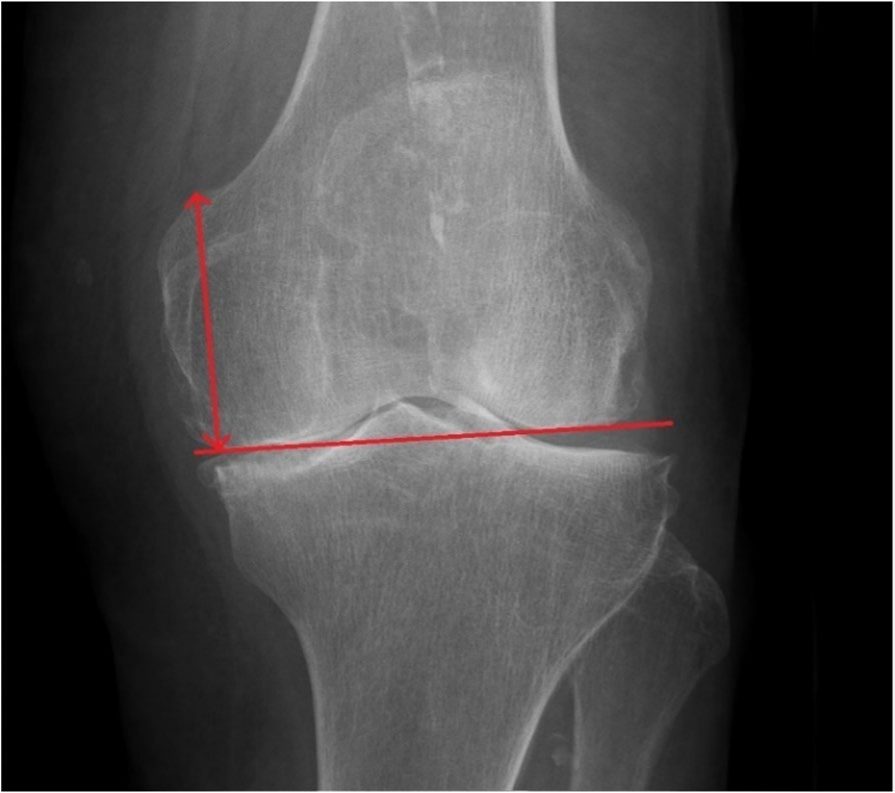
Measurement of preoperative joint line level on the weight-bearing antero-posterior x-ray

**Fig. 2:**
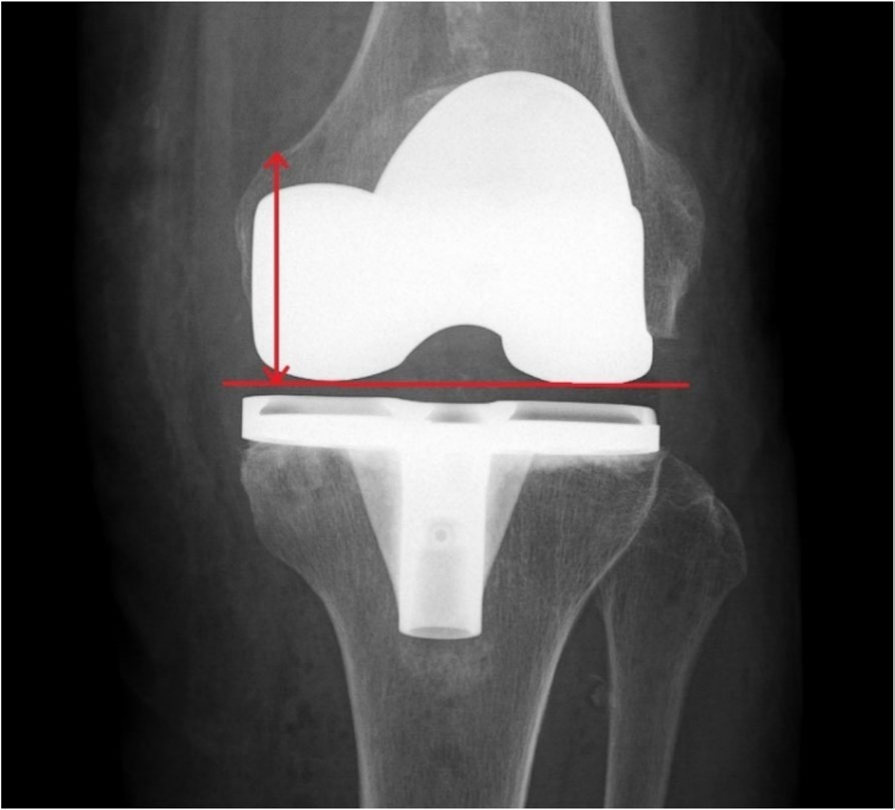
Measurement of postoperative joint line level on the weight-bearing antero-posterior x-ray

Pre- and postoperative standard standing X-rays were performed in the AP and lateral views (Figs. 1 and 2). Difference in joint line level and posterior condylar offset were assessed. The joint line was defined on the anteroposterior radiographs as the lines tangent to the articular surfaces of the medial and lateral femoral condyles. The pre- and post-operative distances between the adductor tubercle of the femur and the joint line were measured (JL), according to the study by Hoffmann et al. [13].

**Fig. 3:**
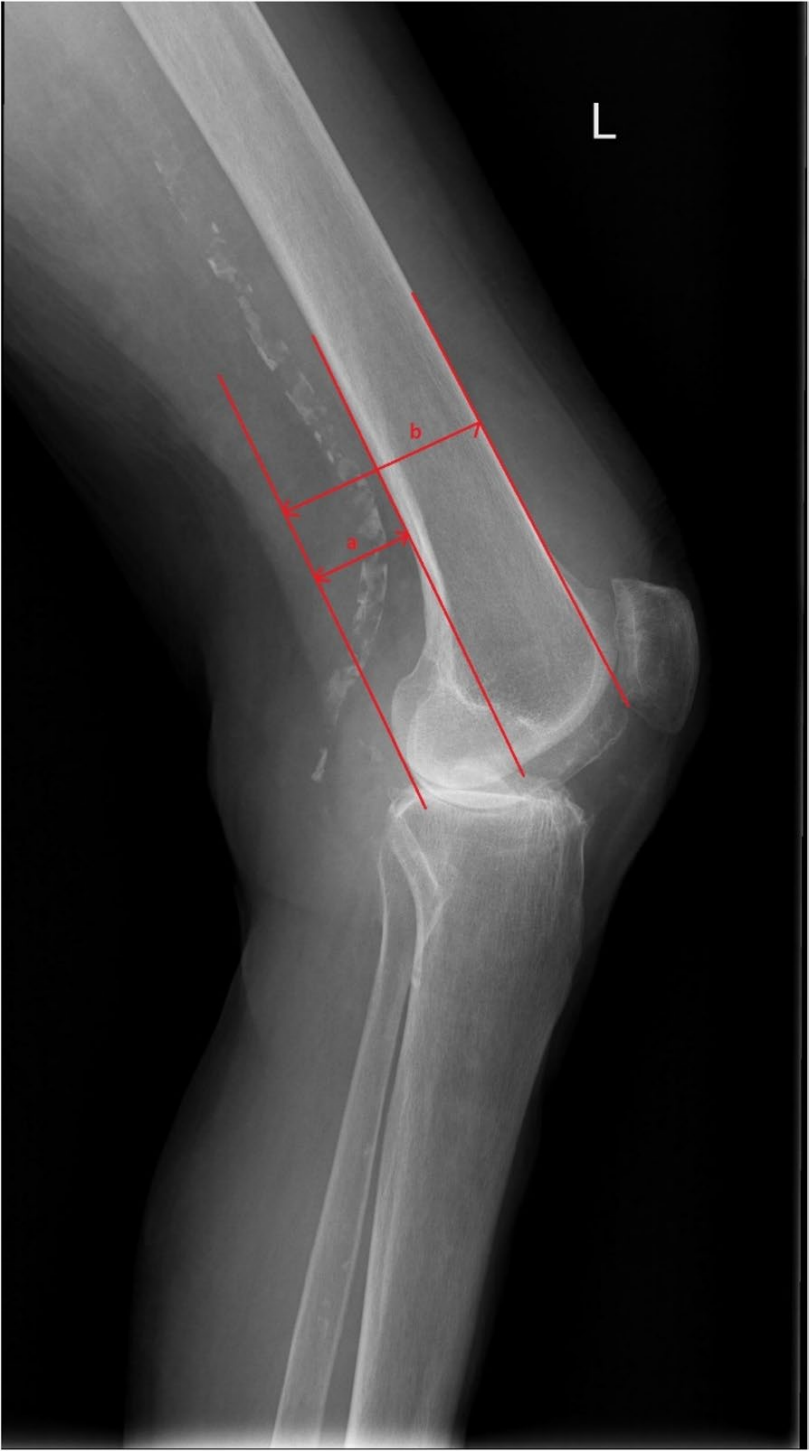
Measurement of preoperative posterior condylar offset ratio (a/b) on standard lateral x-ray

**Fig. 4:**
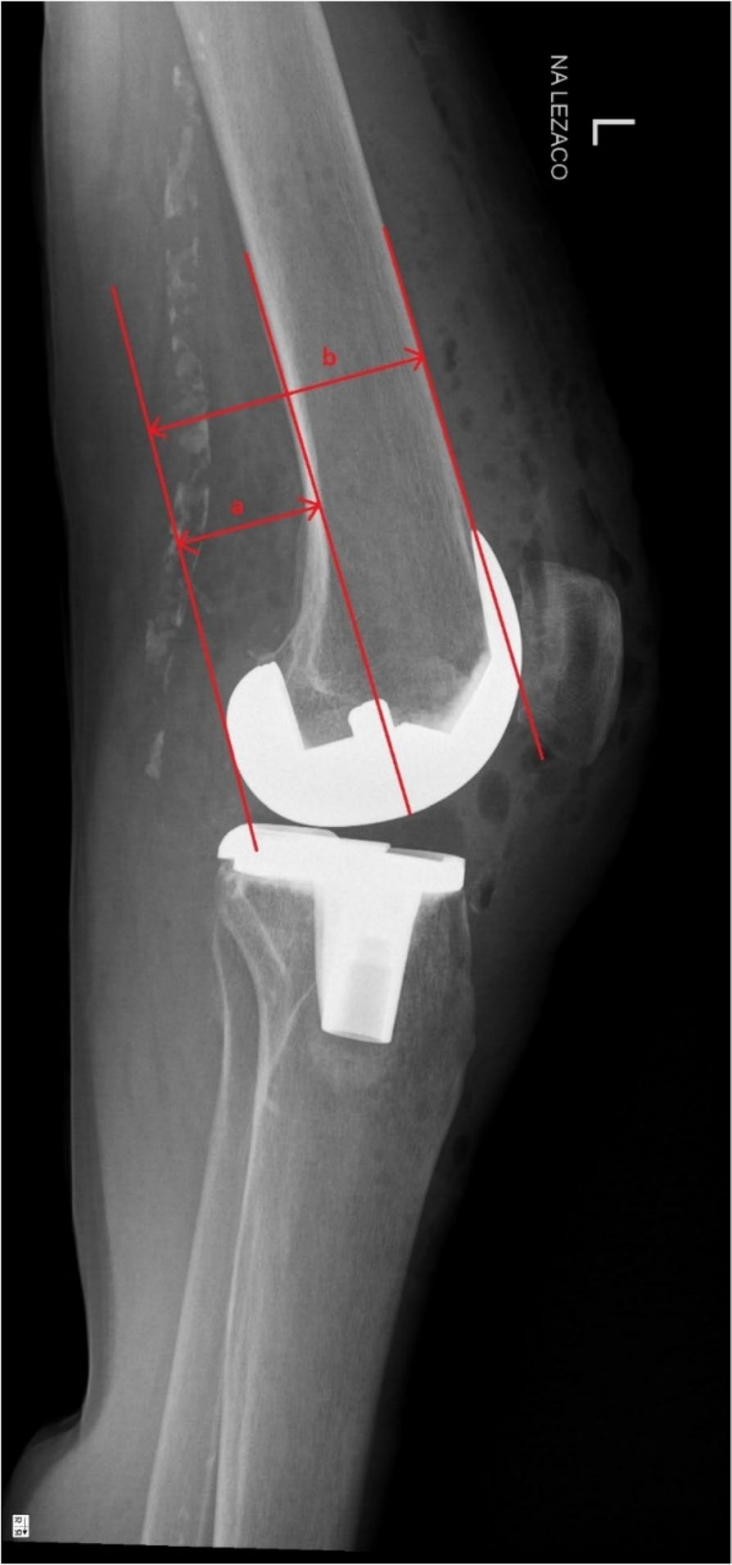
Measurement of postoperative posterior condylar offset ratio (a/b) on standard lateral x-ray

Posterior condylar offset ratio (PCOR) was measured on the lateral radiographs as the ratio between: (1) the distance from the posterior articular surface of the femoral condyles to the tangent to the posterior cortex of the femoral diaphysis and (2) the distance between the posterior articular surface of the femoral condyles and the tangent to the anterior cortex of the femoral diaphysis [14] (Figs. 3 and 4). The cut point of the PCOR change values was + -10%.

All radiographs were measured three times by two independent researchers, and mean values of their results were noted. To avoid potential risk of bias, all data concerning participants was blinded. Mean intra- and interobserver differences in measurements of femoral and tibial components were calculated for all cases. Both Inter- and Intra-rater reliability was estimated using Intraclass Correlation Coefficients (ICC), based on a mean-rating (k = 2), absolute agreement, two-way random effects. All ICC estimates were greater than 0.8.

### Statistical analysis

Potential outliers were identified using the 1.5 IQR rule and investigated individually before deciding whether to include or exclude them from the analysis. All comparisons were performed between independent variables. For comparisons between continuous variables either Student's t-test or Mann's U test were used in accordance to normality of distribution examined with the Shapiro–Wilk test. For comparisons between categorical variables Fisher's exact test was performed. An α value of 0.05 was used to determine the statistical significance of all the analyses. All statistical analyses were conducted using SAS software, Version 9.4 for Windows (SAS Institute Inc., NC, USA).

## Results

Two hundred twenty-five knees were included in the analysis. One hundred one arthroplasties were performed with gap-balancing and 124 with measured resection technique. Baseline characteristics of the participants are depicted in Table 1.

There were significant differences between gap balancing and measured resection groups in change of JL parameter (-2.6 ± 4.1 vs -0.7 ± 4.8, *p* = 0.0019) and ratio of no negative value of JL change – meaning the joint line lowering in favour of measured resection technique (27:74vs 59:65, *p* = 0.0015) and in rate of JL change values exceeding 2 mm. in favour for measured resection group. (10:91 vs 26:98, *p* = 0.028).

In terms of PCOR changes expressed in percentage of initial value (100.8 ± 11.8 vs 101.0 ± 12.5, *p* = 0.8) and rate of restoring PCOR within + -10% of initial measurement (66:35 vs 80:40, *p* = 1), respectively (Table 2).

**Table 2:** Comparison of mean values of joint line elevation between gap balancing and measured resection techniques

		**Gap balancing**	measured resection	***p*-value**
**Mean value of joint elevation (mm)**		-2.6 ± 4.1	-0.7 ± 4.8	** < 0.05**
**Change in the joint line elevation pre- and postoperatively (number of patients)**	** ≥ 0 mm**	27	59	** < 0.05**
	** ≥ 2 mm**	74	65	** < 0.05**
**Change in PCOR + -10% of initial value (% of initial value)**		100.8 ± 11.8	101 ± 12.5	**0.8**

Negative values mean joint line lowering. Comparison of number of patients with joint line elevation with ≥ 0 and ≥ 2 mm between gap balancing and measured resection techniques. Differences in restoring posterior condylar offset ratio (PCOR). Underlined values are considered statistically significant

## Discussion

The most important finding of the presented study is that the results of the measurements suggest that measured resection technique seems to be superior regarding joint line level maintenance. This finding coincides with up-to-date studies. It also suggests that none of the techniques utilized was superior in terms of restoring preoperative PCOR level. There are many implications of joint line elevation. It may lead to anterior knee pain, decreased postoperative knee range of motion, patella-implant or patellar ligament-implant impingement syndromes and correspond to worse knee functional outcomes [15]. Fornalsky et al. found that joint line elevation of 8 mm caused significant decrease in patella-femur contact in 60, 90, 120 angles of flexion [16]. Pseudo-patella baja is another result of joint line elevation. It can cause patello-tibial impingement in deep flexion, increases midflexion instability and amplifies tibio- or patello-femoral forces threatening implant failure [17]. Restoration of posterior condylar offset is crucial for maintenance of proper knee alignment. It directly affects ROM (range of motion) and midflexion stability of the joint [18]. In this study, authors used anatomic implants, which are considered superior in coverage of tibial plateau, which has many benefits [19, 20]. Better coverage decreases loosening and tibial component malrotation rate. It improves femoral component rotation and has a positive effect on patellar traction [21, 22]. We utilized two major TKA techniques which differ in references when it comes to performing bone cuts. The goal of measured resection is to replace removed bone with an implant of the same size and thickness. Measured resection relies on reference points aiming to place the femoral component parallel to the Whiteside or sulcus line or perpendicular to the transepicondylar axis. Another reference is posterior condylar axis (PCA). Properly used, these references provide a rectangular flexion gap, while rectangular extension gap sometimes must be obtained by soft tissue releases. Downsides of measured resection are differences in reference points caused by individual variability and deformations and potential difficulties in recognizing bony landmarks [23, 24]. In the study by ESSKA-EKA it was stated that none of the bony landmarks alone can be considered a perfect reference [25]. Very often it's impossible to predict the gap space following soft tissue releases for extension gap balancing, which might lead to malrotation of femoral components causing coronal instability. On the other hand, there is gap balancing technique where the first goal is to get rectangular gaps in extension and flexion which is achieved by asymmetrical distal femoral cut (extension balance) and setting rotation of the femoral component in order to get a rectangular flexion gap. Among the negatives of gap balancing are postoperative midflexion instability, non-anatomical femoral component rotation, additional space created after PCL removal in PS prosthesis and potential soft tissue imbalance when not all osteophytes are removed before performing cuts [26, 27].

In the most recent meta-analysis by Migliorini et al., the authors conducted review studies comparing measured resection and gap balancing techniques. They stated that gap balancing is associated with joint line elevation, and potentially better functional results. These radiological results correspond with this particular research [6].

The most important finding of presented study is that it suggests MR technique being superior regarding joint line level maintenance. This conclusion agrees with up-to-date studies. It also revealed that none of the techniques utilized was superior in terms of restoring preoperative PCOR level. The reason for such results of this study might be the fact that anatomical TKA designs differ mainly in tibial component horizontal shape but not thickness, so it merely influences posterior femoral cut and joint line level.

The current study has some limitations. Firstly, this is a retrospective matched-cohort study design. To avoid the risk of selection bias, we enrolled a series of consecutive patients and matched them between groups according to age, sex and BMI. Performing the prospective randomized-controlled trial would improve the scientific value of this study. Secondly, this study has not considered data on the degree of joint damage, duration of symptoms, coexisting injuries or other joint pathologies present before surgery that may affect the final outcome. Finally, it is worth noting that despite the fact that the analysed group of patients is quite numerous, a larger number of patients qualified for the study could have an impact on the final results. Nevertheless, the results obtained in the study seem to be quite strong and reliable.

## Conclusions

The results of this study suggest that gap balancing technique with use of anatomic implants seems to be inferior in restoring joint line level in comparison to the measured resection technique. What is more, there is no significant difference in PCO and PCOR between both techniques. However, it was as exact in terms of restoring posterior condyles as the measured resection technique. It is unclear whether such results will lead to worse patient reported outcomes in the future.

## Data Availability

The datasets used and/or analysed during the current study will be available from the corresponding author on reasonable request. Identifying/confidential patient data would not be shared.
